# Pathological pathway analysis in an experimental rheumatoid arthritis model and the tissue repair effect of acupuncture at ST36

**DOI:** 10.3389/fimmu.2023.1164157

**Published:** 2023-05-15

**Authors:** Yue Zhang, Hui Wang, Yi-Nan Gong, Fu-Ming Yang, Shen-Jun Wang, Yang-Yang Liu, Yong-Qing Gui, Fei Xie, Zhi-Fang Xu, Yi Guo

**Affiliations:** ^1^ Research Center of Experimental Acupuncture Science, Tianjin University of Traditional Chinese Medicine, Tianjin, China; ^2^ School of Acupuncture & Moxibustion and Tuina, Tianjin University of Traditional Chinese Medicine, Tianjin, China; ^3^ National Clinical Research Center for Chinese Medicine Acupuncture and Moxibustion, Tianjin, China

**Keywords:** acupuncture, antigen-induced arthritis, collagen-induced arthritis, inflammation, anti-inflammatory, tissue repair

## Abstract

Rheumatoid arthritis (RA) is an autoimmune disease that generally affects the joints. In the face of inflammation-induced cartilage and bone damage, RA treatment remains insufficient. While research evidence indicates that acupuncture can exert anti-inflammatory and analgesic effects, improve the joint function of RA patients, and delay the disease, data on whether it can promote RA repair are lacking. Findings from the present work demonstrated that both the antigen-induced arthritis (AIA) and collagen-induced arthritis (CIA) models can simulate joint swelling of RA. The AIA model was more stable than the CIA model, with a higher incidence of successful arthritis modeling. Moreover, the AIA mice model could simulate the signal molecules and related pathological processes of the autoimmune response in RA, as well as major pathways related to RA and antigen immune response mechanisms. Manual acupuncture (MA) at *Zusanli* (ST36) significantly improved paw redness and swelling, pain, and inflammatory cell infiltration in the joints in AIA mice. The therapeutic effect of MA on AIA is achieved primarily through the regulation of steroid hormone biosynthesis, cell metabolism, and tissue repair processes. MA at ST36 can increase the gene contents of tissue repair growth factors, including *PEG3*, *GADD45A*, *GDF5*, *FGF5*, *SOX2*, and *ATP6V1C2* in the inflammatory side joints of AIA mice, as well as the gene expression of the anti-inflammatory cytokine *IL-10*. In conclusion, acupuncture may alleviate RA in the joints *via* modulating the tissue healing process.

## Introduction

Rheumatoid arthritis (RA) is a chronic systemic autoimmune condition that affects between 0.5% and 1% of the global population (1) and 0.35% of the Chinese population (2). Autoantibodies [rheumatoid factor and anti-citrullinated protein antibody (ACPA)] stimulate fibroblast-like synoviocytes, macrophages, and CD4^+^ cells with pro-inflammatory and tissue-invading effects, triggering a series of cascades that aggravate and perpetuate inflammation (3). Joints are the major sites of involvement in RA; this is associated with synovial hyperplasia, cartilage degeneration, and bone destruction, as well as pain, and eventually loss of function, causing disability and continuous deterioration in the quality of life. In this view, preventing disease progression and joint damage has emerged as a crucial clinical goal of RA treatment. Cytokines contribute to the pathogenesis of bone and cartilage disease by causing abnormal differentiation and function in bone and cartilage cells. Immunosuppressive treatment has been developed over many years as a result of the in-depth understanding of the interaction network of key cytokines in RA pathogenesis. However, given the complex network, targeting a single cytokine can only eliminate or inhibit specific pro-inflammatory cytokines, cell surface receptors, and cell functions and cannot correct the imbalance of multiple immune cells. While disease-modifying anti-rheumatic drugs and steroidal anti-inflammatory drugs can inhibit RA inflammation, long-term usage is frequently associated with poor tolerability, liver damage, osteoporosis, diabetes, and other side effects (4, 5). However, non-steroidal anti-inflammatory drugs used to alleviate pain do not prevent joint damage or delay the progression of RA. Therefore, how to reduce the side effects of treatment while alleviating inflammation and pain and delaying bone destruction is a challenge that remains to be solved in the field of medicine.

The rodent model is a powerful and widely used tool for assessing the pathogenesis and therapeutic mechanisms of RA. While no animal model can fully reflect joint and systemic features, immunological features, and genetic components of human RA, rodent models do mirror some of the hallmarks of human RA in etiology and/or disease. Laboratory-induced arthritis models such as antigen-induced arthritis (AIA) and collagen-induced arthritis (CIA) are based on different immune pathways and show distinct pain-like behaviors and pathological features due to different arthritogens (6, 7). As such, there is a need to develop animal models that are suited for studying the biology and molecular mechanisms of specific interventions in animal models with diseases and/or pathogenesis comparable to human RA.

Acupuncture is a popular complementary and alternative therapy for RA in China, and the World Health Organization recommends it for treating a wide range of inflammatory conditions, notably inflammatory pain. Acupuncture has demonstrated benefits in the treatment of RA by relieving inflammatory reactions such as burning pain, redness, swelling, and temperature changes. Several high-quality evidence-based medical research has also validated the anti-inflammatory effects of acupuncture. A systematic review of nearly 1,000 patients found that acupuncture can significantly relieve pain, morning stiffness, swelling, and other RA symptoms, as well as improve their quality of life (8). Findings from the most recent network meta-analysis of randomized controlled trials of RA treated with acupuncture demonstrated that electroacupuncture had the best effect on improving joint range of motion, and manual acupuncture (MA) was superior to electroacupuncture in lowering the levels of C-reactive protein, erythrocyte sedimentation rate, and RF and with a better anti-inflammatory effect (9). In our previous study, we found that acupuncture at *Zusanli* (ST36) can activate the endogenous regulatory system of the body to combat RA inflammation. It also regulates M1/M2 macrophage homeostasis by inhibiting the increase in macrophage M1/M2 ratio. Results demonstrated that it suppresses inflammatory response by inhibiting pro-inflammatory cytokines [tumor necrosis factor (TNF)-α, interleukin (IL)-1β] (10). Compared with inhibitors that target individual cytokines, acupuncture can concurrently regulate the level of multiple cytokines and chemokines. However, the regulatory mechanism of acupuncture is complex, and the action pathway related to the acupuncture-driven inhibition of joint inflammatory response and improvement of dysfunction remains unclear.

**Figure 1:**
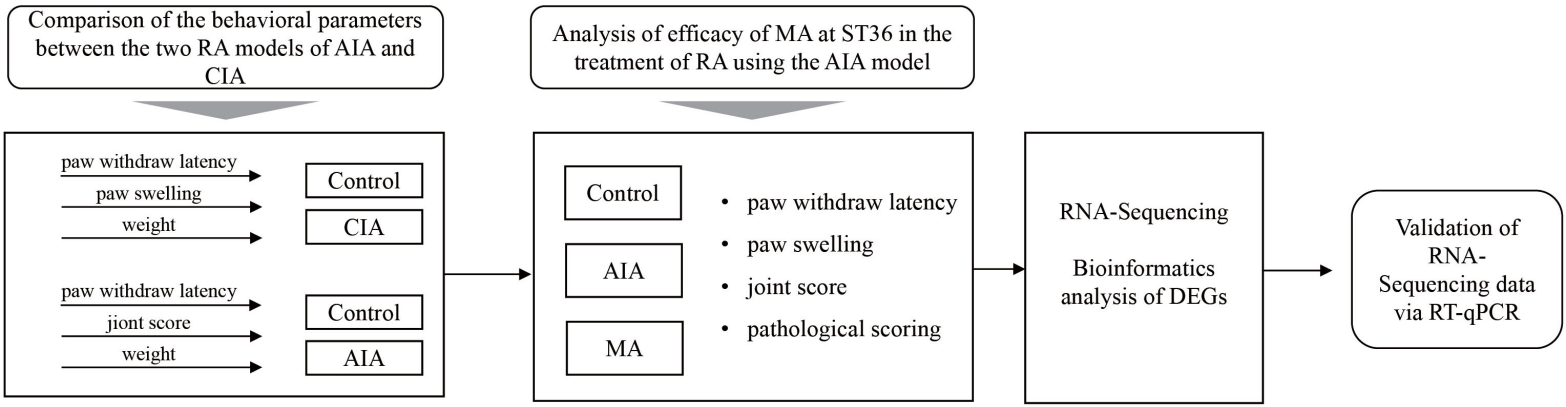
The flowchart of the entire study.

In the present investigation, we selected a relatively stable RA mouse model by comparing the behavioral parameters of AIA mice and CIA mice and further defined the analgesic and anti-inflammatory effects of acupuncture at bilateral ST36 on AIA mice. In addition, we evaluated the mechanical characteristics of the AIA model and the potential action pathways and molecular targets of acupuncture for anti-inflammatory and tissue repair using RNA sequencing, bioinformatics analysis, and molecular biology experiments (**Figure 1**). Our findings provide an experimental basis for elucidating the effect and mechanism of acupuncture in RA treatment.

## Materials and methods

### Animals

Healthy male BALBC/6 mice and DBA/1 mice (6–8 weeks old), weighing 20–22 g, were bought from the experimental animal center of Beijing Wei Tong Li Hua Experimental Animal Technology Co., Ltd. (Beijing, China), license number: SCXK [Beijing] 2014-0004. All mice were housed in cages under controlled conditions at 25°C ± 1°C and 55% ± 5% humidity. The mice were exposed to a 12-h light/12-h dark cycle and allowed free access to food and water. All animal studies were conducted in line with the guidelines on the care and use of laboratory animals established by the Ministry of Science and Technology of China. The animal experiments were approved by the Animal Care and Use Committee of Tianjin University of Traditional Chinese Medicine (permit # TCMLAEC2012009).

### Experimental design

#### Experiment 1: inflammation and associated nociceptive response in the two RA models of AIA and CIA

**Figure 2:**
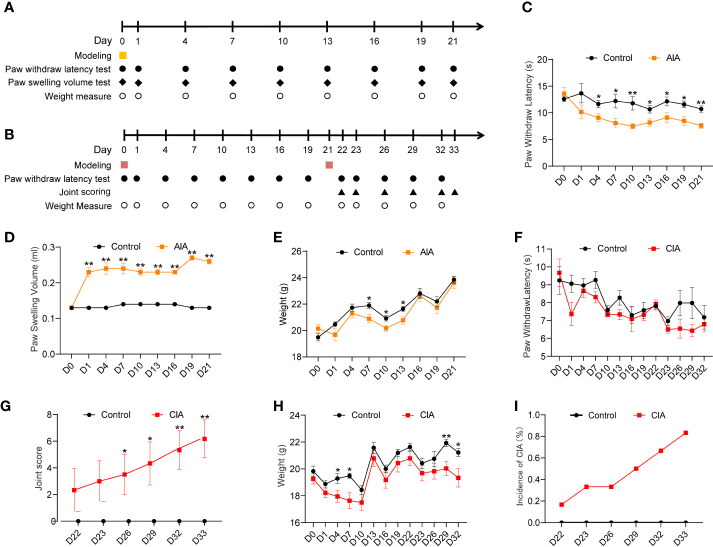
Inflammation and associated nociceptive responses in mice for the antigen-induced arthritis (AIA) and collagen-induced arthritis (CIA) models. **(A)** Flowchart illustrating the AIA modeling process and measurement of the effect index. Disposition is represented by different symbols: ▪ refers to complete Freund’s adjuvant (CFA) or saline planter injection, • indicates the paw withdrawal latency (PWL) test, ▴ represents the paw swelling volume test, and ○ represents the weight (*n* = 6 per group). **(B)** An illustration of the CIA modeling process and measurement of effect index. Disposition is represented by different symbols: ▪ indicates bovine type II; collagen emulsion or saline tail root injection, • represents the PWL test, ▴ shows the clinical severity score of arthritis evaluation, and ○ refers to weight (*n* = 6 per group). The inflammation and associated nociceptive responses of inflamed paws and joints from AIA mice and PWL in **(C)**, paw swelling in **(D)**, and weight in **(E)**. The inflammation and associated nociceptive responses of inflamed paws and joints from CIA mice and PWL in **(F)**, joint score in **(G)**, and weight in **(H)**. **(I)** Incidence of CIA during the 21 days. Data are presented as the mean ± SD. ^*^ *p* < 0.05, ^**^ *p* < 0.01 *vs.* control.

BALB/C mice were randomly divided into two groups: the control group and the AIA group (*n* = 6 per group). On day 0, complete Freund’s adjuvant (CFA) or saline was injected into the right plantar (i.pl.). On day 0 and days 1–21 (every 3 days), the paw withdrawal latency (PWL) was tested for each mouse. The swelling degree of the right hind paw (foot swelling and joint score in the paw) was measured and evaluated, and body weight (**Figure 2A**) was recorded. The DBA/1 mice were randomly divided into two groups: the control group and the CIA group (*n* = 6 per group). The first immunization was initiated by the subcutaneous injection of collagen emulsion 100 μl into the tail root of DBA/1 mice (2–3 cm) on day 0. On day 21, 100 μl of bovine type II collagen emulsion was subcutaneously injected into the tail root to induce secondary immunity. Subsequently, we measured the PWL and body weight of mice on day 0, days 1–21 (every 3 days), day 22, and days 23–32 (every 3 days). The joint score (**Figure 2B**) of the right hind paw was recorded on day 22, day 33, and days 23–32 (every 3 days).

#### Experiment 2: analysis of the behavioral changes in AIA mice treated with manual acupuncture and the differential expression of ankle joint genes after manual acupuncture treatment

**Figure 3:**
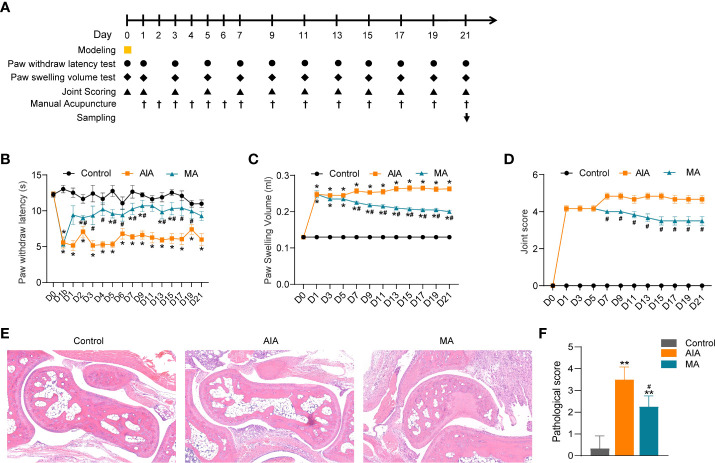
The anti-inflammatory and anti-nociceptive effects of ST36 manual acupuncture (MA) on inflamed paws and joints of mice with antigen-induced arthritis (AIA). **(A)** Flow chart showing the acupuncture treatment procedure and the effect index measurement. ▪ indicates CFA or saline planter injection, • indicates the PWL test, ♦ indicates the paw swelling volume test, ▴ represents joint score evaluation, ^†^ represents MA administration, and ↓ represents sacrifice and tissue collection. **(B–D)** The effect of MA treatment on CFA-induced paw and PWL in **(B)**, paw swelling in **(C)** and joint score in **(D)** (*n* = 6 per group). **(E)** Histological analyses of right ankle joints (×100) stained by H&E, and different degrees of erosion in the articular cartilage and subchondral arthritis specimen from the AIA and MA groups (*n* = 3–4 per group). **(F)** Quantitative analysis of the pathological assessment of the three groups. Data are presented as the mean ± SD. *p < 0.05 vs. control. ^**^ *p* < 0.01 *vs.* control. ^#^ *p* < 0.05 *vs.* AIA.

In the subsequent experiment, BALB/C mice were randomly divided into three groups: the control group, the AIA group, and the MA group (*n* = 6 per group). On day 0, mice were injected with CFA or saline into the right plantar (i.pl.). On day 0 and days 1–21 (every 3 days), the PWL of the inflamed side was measured, and the swelling degree of the right hind paw (foot swelling and joint score) was determined. MA treatment was conducted on days 1–7 (once a day) and days 8–21 (every other day). On day 21, the inflamed ankle joints (**Figure 3A**) were collected to further evaluate the pathological damage (*n* = 3–4 per group) and perform RNA sequencing (*n* = 6 per group) and RT-qPCR (*n* = 6 per group).

### Establishment of AIA and CIA model

The AIA mouse model was established through intradermal injection of 0.05 ml of CFA (Sigma, San Diego, CA, USA) into the right plantar of mice on day 0. For mice in the control group, 0.05 ml of 0.9% normal saline was injected into the right plantar. Bovine type II collagen emulsion was prepared by mixing 2 mg/ml of bovine type II collagen (Chondrex, USA) with 1 mg/ml of complete Freund’s adjuvant. The solution was injected subcutaneously at the base of the DBA/1 mouse tail (100 μl per mouse) on day 0. At 21 days after the first immunization, the mice were injected with bovine type II collagen emulsion subcutaneously as a booster immunization (100 μl/only)(11). Mice in the control group were injected with 0.9% saline solution 100 μl at the base of the DBA/1 mouse tail.

### Manual acupuncture treatment

Mice in the MA group were immobilized with a soft cloth. Disposable acupuncture needles (diameter = 0.16 mm, length = 7 mm, Zhongyan Taihe, Beijing, China) were vertically inserted into bilateral ST36, in the muscle groove of the fibula head approximately 2 mm at the outer lower knee joint of mice to a 3-mm depth. After deqi, twirling manipulation was performed, through the mild reinforcing–attenuating method. The acupuncture frequency was controlled using a metronome, set at a frequency of 180 times/min. The twirling manipulation lasted for 2 min and was performed once every 5 min, for 4 repeats, with a total time of 28 min.

### Measure of thermal nociceptive threshold

For BALB/C mice, PWL was measured using a plantar tester (37370, Ugo Basile, Italy), which was considered to be the thermal nociceptive threshold indicator. The mice were acclimatized to an indoor environment for 30 min before measurements were recorded. For each mouse, measurements were taken three times, at a 5-min time interval, and the average value of PWL was determined. A hot/cold plate (35150, Ugo Basile, Italy) was used to measure the thermal nociceptive threshold of each CIA mouse. The mice were acclimatized to the indoor environment for 30 min before measurements. The hot plate temperature was set to 55°C ± 0.2°C. The time of the first appearance of pain behavior (foot lifting, jumping, licking, or climbing the wall) was measured as PWL. For each mouse, the measurements were conducted three times at 5 min intervals, and the average value was determined.

### Determination of the ankle and paw swelling

The swelling of the ankle joint and paw was measured using a plethysmometer (Ugo Basile, Italy) device. For each mouse, measurements were conducted three times and the average value was calculated.

### Joint score

Erythema and swelling of the ankle, foot, and digits or ankylosis of the limb were determined on the basis of 0–4 scores (12). The score for severity per animal was calculated by adding the results from all paws. The total score ≥ 4 indicated that the model was successful. The incidence of CIA = (the number of successful modeling/total) * 100%.

### Hematoxylin and eosin staining

On day 21, after acupuncture intervention, thermal nociceptive threshold and foot swelling measurement and joint scores were recorded at approximately 1.5 h after acupuncture. The mice were weighed and anesthetized by intraperitoneal injection of 5% chloral hydrate (0.10 ml/10 g). The inflamed ankle joint of the mice was recorded, and the skin of the ankle joint was removed using ophthalmic scissors. The specimens were rinsed with PBS and oct-embedded. They were then sectioned to 5.0 mm thickness using a Lecia frozen slicer (1950) and stained with hematoxylin and eosin (H&E). Finally, a biomicroscope (Nikon E11clipse E100, Japan) was used to examine the histological sections and take photographs (Nikon DS-U3, Japan). Pathological scores in the range of 0–5 indicate five different levels, from no inflammation to synovium highly infiltrated with many inflammatory cells (13).

### RNA sequencing analysis

The ankle joint was collected as previously described. The ankle joint was cleaned with PBS and frozen in liquid nitrogen and kept in a refrigerator at −80°C for further RNA sequencing. Total RNA samples were extracted from the control, AIA, and MA ankle joint samples using Rneasy Mini Kit (Qiagen, Germany). Paired-end libraries were synthesized using the TruSeq^®^ RNA Sample Preparation Kit (Illumina, USA) in line with the TruSeq^®^ RNA Sample Preparation Guide. Library construction and sequencing were performed by the Shanghai Biotechnology Corporation. Differentially expressed genes (DEGs) were identified using edgeR (14). The false discovery rate (FDR) was used to determine the significance level at a *p-*value in multiple tests (15, 16). Additionally, the fold changes were calculated based on the FPKM in each sample. The DEGs were selected based on FDR 0.05 and fold change 2 as threshold criteria. The functions of the DEGs were investigated *via* Gene Ontology (GO) analysis. According to biological processes and molecular activities, GO analysis classifies genes into hierarchical categories and can reveal regulatory networks of various genes (http://www.geneontology.org). The retrieved GO terms were sorted based on size in descending order based on the value of the enrichment factor and the top 30 pathways. The Kyoto Encyclopedia of Genes and Genomes (KEGG, http://www.genome. Jp/kegg/) pathway analysis was performed to determine pathways associated with the DEGs. The KEGG enrichment was calculated similar to the calculation for GO analysis. Based on the results of the preceding study of DEGs from various groups, the protein–protein interaction (PPI) network was constructed using the STRING database.

### Real-time quantitative PCR

RNA samples were extracted by Shanghai Biotechnology Corporation and verified by RT-qPCR. The total RNA was reverse-transcribed using the 5 PrimeScript RT Master Mix (Takara Bio, Inc., Otsu, Japan) in accordance with the manufacturer’s instructions. RT-qPCR was then conducted using SYBR Premix Ex Taq™ (Takara Bio, Inc., Otsu, Japan) on an ABI 7500 machine (Applied Biosystems, Foster City, CA, USA) following the manufacturer’s instructions. The primers used for PCR were designed by Dalian Bao Biotechnology Co. Ltd. and are presented in **Table 1**. The relative gene expression was calculated using the 2^(−Δ Δ Ct)^ technique.

**Table 1:** The list of primers.

Gene name	Accession (gene)	Forward primer	Reverse primer
*IL10*	NM_010548.2	GAAGCATGGCCCAGAAATCAAG	CACAGGGGAGAAATCGATGACA
*Calm4*	NM_020036.4	CAAGAACCTCCCAGAGAAGGAC	TGTGCCCTTTCTTGTACTTCTCT
*GDF5*	NM_008109.3	GGGCAAAGCATCTTCAAAAGCA	ACAGGGAGAGCATGTATTCGTG
*ATP6V1C2*	NM_001159632.1	AGTCTAACCTGTCCCACAACAC	GTATCGAGTTTCCCCAGCTCAT
*Gadd45a*	NM_007836.1	CTCTGCAGATCCATTTCACCCT	GTCGTTCTCCAGTAGCAGCAG
*FGF5*	NM_001277268.1	TCGGAACATAGCAGTTTCCAGT	TTTGGCTTAACACACTGGCTTC
*SOSTDC1*	NM_025312.3	TGAATCAAGCCAGGAATGGAGG	CCGTCCGAAATGTATTTGGTGG
*PEG3*	NM_008817.2	CGGCTCCAGTACTTTGTTCCAT	GGACAAGAAGCCTCAGAATCCA
*SOX2*	NM_011443.4	GCGGAGTGGAAACTTTTGTCC	TCCTTCTTCATGAGCGTCTTGG
*Icam1*	NM_010493.3	CAGAAGTTGTTTTGCTCCCTGG	ACTCCTCAGTCACCTCTACCAA

### Statistical analysis

The Statistical Package for the Social Sciences (SPSS) Version 19.0 (SPSS Inc., Chicago, IL, USA) was used to analyze the data. The multivariate ANOVA test was employed to determine the differences between groups, and several measurements were recorded at different time points (such as paw volume and PWL). One-way ANOVA was used to compare data from the same time point in multiple groups. *Post hoc* tests of least significant difference (LSD) were adopted if the variances of all populations were equal, otherwise Dunnett’s T3 method was used. *p <*0.05 was considered statistically significant. Graphs were created using the GraphPad Prism software (GraphPad, San Diego, CA, USA).

## Results

### Establishment of the experimental RA models

The RA models of AIA and CIA were established in this study. The AIA group demonstrated acute inflammation (redness, swelling, and heat pain) 24 h following the CFA injection, and the right paw displayed a significant reduction in thermal nociceptive threshold (represented as the PWL). The thermal nociceptive threshold of the AIA group decreased significantly (*p* < 0.01) on day 4 and lasted until day 21 compared with the saline-injected group (**Figure 2C**). Similarly, as shown in **Figure 2D**, the AIA mice exhibited sustained swelling of the inflamed paws and ankles on day 1 after modeling, peaked on day 19, and continued until day 21 (*p* < 0.01) (**Figure 2D**). During the assessment period, the body weight of mice in the AIA group was significantly lower than that of the control group on days 7, 10, and 13 following CFA injection (*p* < 0.05), but there was no difference between the two groups after day 13 (**Figure 2E**). The thermal nociceptive thresholds of the two groups were compared following primary immunization of CIA mice. The changes in the two groups showed a fluctuating decreasing trend; however, the difference in the thermal radiation pain threshold was not statistically significant between the two groups (**Figure 2F**). According to the joint score of the CIA group and the control group, mice in the CIA group displayed evident swelling of the joint on the inflammatory side on day 26, 4 days after the second immunization (day 22) (*p* < 0.05), persisted through day 33 and peaked on day 33 (*p* < 0.01), whereas the joint score of the control group was 0 during the assessment period (**Figure 2G**). The overall incidence of successful CIA modeling was 83.3% (**Figure 2I**). Although no significant difference in body weight was found between the CIA and control groups before modeling, the body weight of both groups showed an upward trend over the monitoring period. Generally, the weight of the body of CIA mice was lower compared with the control group. There were significant differences in body weight between the two groups on days 4, 7, 29, and 32 (*p* < 0.05) (**Figure 2H**). The aforementioned findings demonstrate that this study successfully constructed the AIA and CIA types of experimental RA models. The AIA model is built faster and has a more consistent degree of inflammatory pain and swelling. While the CIA model takes a longer time to construct, has evident inflammatory symptoms and foot swelling, and has a lower replication rate, the performance of inflammatory pain is unstable and makes it difficult to observe the analgesic and anti-inflammatory effects of MA in the next experiment. In this view, we selected the AIA model to investigate the impact of MA.

### Assessment of the pathogenesis of the AIA model mice using bioinformatics analysis

**Figure 4:**
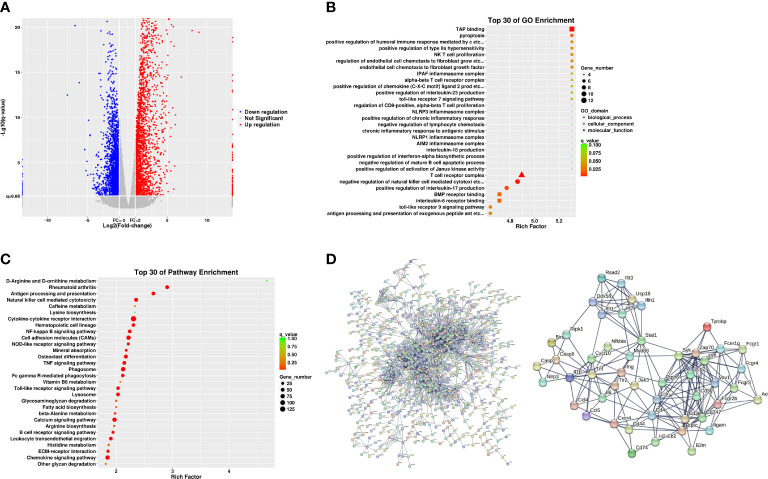
Analyses of the expression and enrichment of genes in joint specimens from AIA mice. **(A)** Scatter plots of differentially expressed genes (DEGs) in the control group and AIA groups. Each probe is represented by a point with red and blue points showing up- and downregulated genes based on log2 FC > 2 cutoff. **(B)** GO analyses of DEG results. **(C)** KEGG analyses of DEG results. **(D)** PPI network analyses of DEG results.

The molecular mechanisms of the AIA model were investigated using RNA sequencing. In comparison with the control group, 4,845 DEGs were identified for the ipsilateral ankle joint, of which 2,644 were upregulated and 2,201 were downregulated (**Figure 4A**). The GO analysis classifies genes into three functional groups: biological process (BP), cellular component (CC), and molecular function (MF). The most significantly enriched BP terms included negative regulation of natural killer cell-mediated cytotoxicity, antigen processing and presentation of exogenous peptide antigen *via* major histocompatibility complex (MHC) class I, and positive regulation of humoral immune response mediated by circulating immunoglobulin and natural killer (NK) T-cell proliferation. CC terms had the highest significant enrichment in the T-cell receptor complex, ice protease-activating factor (IPAF) inflammasome complex, and alpha-beta T-cell receptor complex. MF terms were most significantly enriched in transporter associated with antigen processing (TAP) binding, bone morphogenic protein (BMP) receptor binding, and IL-6 receptor binding (**Figure 4B**). According to the KEGG pathway enrichment analysis, pathways associated with RA inflammatory process were enriched to rheumatoid arthritis and osteoclast differentiation, with cytokine–cytokine receptor interaction, cell adhesion molecules (CAMs), antigen processing and presentation, natural killer cell-mediated cytotoxicity, and other pathways related to antigen immune response being the most significantly enriched. The enriched intracellular signal pathways include the calcium signaling pathway, nuclear factor kappa-B (NF- κB) signaling pathway, TNF signaling pathway, Toll-like receptor signaling pathway, and chemokine signaling pathway (**Figure 4C** and **Table 2**). The PPI network built from the STRING database revealed that proteins encoded by the DEGs had complex interactions. The PPI network yielded 801 proteins and 1,997 edges (required score > 0.9), and the first 49 hub genes (degree > 15) (**Figure 4D** and **Table 3**) were listed. Most of these genes were linked to T-cell surface glycoproteins *(Cd4*, *Cd247*, *Cd3g*, *Cd3e*, *Cd44*), interferon signaling (*Stat1*, *Ifng*, *Irf7*, *Ifit3*, *Rsad2*, *Ifih1*, *Usp18*), cytokines and inflammatory response (*Tnf*, *Il1b*, *Il6*, *Cxcl10*, *Cxcr4*, *Ccl5*, *Ccl4*), protein tyrosine kinase (*Ptprc*, *Lck*, *Ptpn6*, *Jak3*, *Tyrobp*, *Vav1*), and apoptosis (*Casp8*, *Casp1*). Furthermore, the expression of the aforementioned hub genes was upregulated following CFA injection. These data demonstrated that AIA activates the processes linked to innate and adaptive immunity and AIA mice can simulate the signal molecules and related pathological processes of an autoimmune response in RA.

**Table 2:** KEGG enrichment of DEGs in the AIA and control groups.

Pathway	*Q* value	Different genes	Upregulated genes	Downregulated genes	Rich factor
Rheumatoid arthritis	<0.01	51	*Ccl3*, *Ccl20*, *Tnfsf13*, *Atp6v1a*, *Il23a*, *Icam1*, *Atp6v0e*, *Ctsl*, *Atp6v1b2*, *H2-DMb1*, *Il1a*, *Il1b*, *Fos*, *H2-Ab1*, *Atp6v0c*, *Atp6v1g2*, *Tcirg1*, *Tnf*, *Ccl2*, *Il17a*, *Tnfsf13b*, *Acp5*, *Tnfrsf11a*, *Ccl5*, *Cd80*, *H2-DMb2*, *Ccl12*, *Tlr2*, *Itgb2*, *Itgb2l*, *Itgal*, *H2-DMa*, *Csf2*, *Tnfsf11*, *Atp6v0b*, *Atp6v0d2*, *Ifng*, *Mmp3*, *Ltb*, *Ctsk*, *Atp6v1c1*, *Il18*, *H2-Aa*, *H2-Eb1*, *Cxcl5*, *Cd86*, *Il6*, *Il15*	*Atp6v0a4*, *Tgfb2*, *Atp6v1c2*	2.902
Osteoclast differentiation	<0.01	61	*Mapk13*, *Ncf4*, *Cyba*, *Junb*, *Mitf*, *Cybb*, *Gm14548*, *Tyrobp*, *Pik3r5*, *Trem2*, *Socs1*, *Lcp2*, *Ncf2*, *Ifnar2*, *Nfkbia*, *Il1a*, *Il1b*, *Fos*, *Pira2*, *Csf1r*, *Spi1*, *Pik3cd*, *Stat1*, *Tnf*, *Acp5*, *Tnfrsf11a*, *Fosl1*, *Lck*, *Pik3cb*, *Socs3*, *Fcgr4*, *Pirb*, *Stat2*, *Fcgr2b*, *Oscar*, *Fosl2*, *Blnk*, *Btk*, *Sirpb1b*, *Fcgr1*, *Nfkb2*, *Syk*, *Ifngr2*, *Tnfsf11*, *Ifng*, *Ncf1*, *Sirpa*, *Ctsk*, *Ifnar1*, *Gm15448*, *Sirpb1a*, *Plcg2*, *Gm9733*, *Fcgr3*, *Lilra6*, *Relb*, *Irf9*, *Pik3cg*	*Mapk12*, *Tgfb2*, *Tnfrsf11b*	2.173
Cytokine–cytokine receptor interaction	<0.01	131	*Ccl3*, *Ccl20*, *Cxcl13*, *Il21r*, *Il1r2*, *Ccr1*, *Il2*, *Ifnlr1*, *Flt3*, *Cxcr6*, *Tnfsf14*, *Il12b*, *Cd27*, *Ccr7*, *Il10ra*, *Cxcr4*, *Tnfsf13*, *Il23a*, *Il4ra*, *Tnfrsf9*, *Il12rb1*, *Cxcl11*, *Ccr4*, *Fas*, *Ccr9*, *Ifnar2*, *Il10*, *Ccl22*, *Il21*, *Tnfrsf8*, *Fasl*, *Il1a*, *Il1b*, *Ccl6*, *Il12rb2*, *Ccl8*, *Il2rb*, *Il7r*, *Cd40lg*, *Il1rap*, *Cxcl10*, *Relt*, *Il10rb*, *Csf1r*, *Ccr5*, *Csf2rb*, *Il17ra*, *Tnfsf4*, *Hgf*, *Tnf*, *Ccl2*, *Il17a*, *Tnfsf13b*, *Cxcl1*, *Cxcl16*, *Tnfrsf11a*, *Csf2ra*, *Tnfrsf13b*, *Ccl5*, *Osm*, *Ccl4*, *Tnfsf15*, *Ccl12*, *Tnfrsf18*, *Tnfrsf14*, *Cxcr3*, *Csf3*, *Xcl1*, *Cd40*, *Crlf2*, *Csf2rb2*, *Il3ra*, *Il15ra*, *Il2rg*, *Cxcr2*, *Cxcr1*, *Csf2*, *Ccl9*, *Ccl7*, *Ifngr2*, *Tnfsf11*, *Csf3r*, *Xcr1*, *Ifng*, *Ltb*, *Cd70*, *Lta*, *Tnfrsf21*, *Ifnar1*, *Il18rap*, *Tnfrsf1b*, *Il18*, *Cxcl9*, *Cxcl5*, *Tnfsf10*, *Cxcl2*, *Il18r1*, *Il13ra1*, *Il6*, *Ccr8*, *Il6ra*, *Ccr2*, *Tnfsf8*, *Tnfsf9*, *Tnfrsf4*, *Il23r*, *Il15*, *Il2ra*	*Acvr2b*, *Ccl24*, *Cntf*, *Ctf1*, *Edar*, *Eda2r*, *Egf*, *Thpo*, *Tnfrsf19*, *Il11ra2*, *Gdf5*, *Tnfrsf17*, *Bmp7*, *Cntfr*, *Ghr*, *Cxcr5*, *Lepr*, *Amhr2*, *Tgfb2, Lep*, *Il17b*, *Eda*, *Tnfrsf11b*	2.307
Cell adhesion molecules (CAMs)	<0.01	78	*Cd8b1*, *Vcam1*, *Tigit*, *Cd274*, *Itgam*, *Icam1*, *Cd22*, *Gm11127*, *Pdcd1lg2*, *Sdc3*, *Selplg*, *H2-DMb1*, *Cd6, Ptprc*, *H2-Ab1*, *H2-Bl*, *Pdcd1*, *Itgb7*, *Cd40lg*, *H2-M2, H2-Q7*, *Sele*, *H2-Q2*, *H2-T23*, *H2-M3*, *Sell*, *Cd2*, *Selp*, *Itga4*, *Gm8909*, *Alcam*, *Cd80, Lrrc4*, *Pvrl2*, *H2-D1*, *H2-DMb2*, *H2-T22*, *Itgb2*, *Itgb2l*, *H2-T24*, *H2-Q10*, *Cd40*, *Itgal*, *H2-DMa*, *H2-Q4*, *Glycam1*, *Siglec1*, *Spn*, *H2-Q6*, *Cd4*, *Cd8a*, *H2-Q1*, *H2-K1*, *H2-Aa*, *H2-Eb1*, *Sdc1*, *Gm7030*, *Cd86*, *H2-T10*	*Cntn2*, *Cdh15*, *Ncam1*, *Cldn19*, *Cldn1*, *Vtcn1*, *Cldn9*, *Ntng1*, *Ncam2*, *Cdh4*, *Cldn22*, *Negr1*, *Mpz*, *Madcam1*, *Itga8*, *Lrrc4c*, *Ocln*, *Cntnap2*, *Cldn3*	2.220
Antigen processing and presentation	<0.01	49	*Ctss*, *Cd8b1*, *Cd74*, *Ifi30*, *Tap2*, *Gm11127*, *Ctsl*, *H2-DMb1*, *H2-Ab1*, *Klrd1*, *H2-Bl*, *Klrc1*, *H2-M2*, *H2-Q7*, *H2-Q2*, *H2-T23*, *Psme2*, *H2-M3*, *Tnf*, *Gm8909*, *Ciita*, *Klrc3*, *H2-D1*, *H2-DMb2*, *H2-T22*, *Tap1*, *H2-T24*, *H2-Q10*, *Psme1*, *H2-DMa*, *Psme2b*, *Ctsb*, *H2-Q4*, *Tapbp*, *Pdia3*, *Lgmn*, *H2-Q6*, *Ifng*, *Cd4*, *B2m*, *Cd8a*, *H2-Q1*, *H2-K1*, *H2-Aa*, *H2-Eb1*, *Gm7030*, *H2-T10*	*Hspa1a*, *Hspa1l*	2.659
Natural killer cell-mediated cytotoxicity	<0.01	59	*Bid*, *Prkcb*, *Vav1*, *Zap70*, *Tyrobp*, *Pik3r5*, *Icam1*, *Lcp2*, *Fas*, *Ifnar2*, *Klra7*, *Fasl*, *Cd48*, *Klrd1*, *Klrc1*, *Ptpn6*, *H2-T23*, *Pik3cd*, *Cd244*, *Rac2*, *Hcst*, *Raet1e*, *Cd247*, *Tnf*, *Sh2d1b1*, *Lck*, *Sh3bp2*, *Pik3cb*, *H2-D1*, *Fcgr4*, *Fcer1g*, *Sh2d1a*, *Klrk1*, *Itgb2*, *Itgb2l*, *Gzmb*, *Vav3*, *Itgal*, *Lat*, *Ulbp1*, *Csf2*, *Syk*, *Ifngr2*, *Ncr1*, *Casp3*, *Prf1*, *Ifng*, *Klrb1c*, *Raet1d*, *Ifnar1*, *H2-K1*, *Plcg2*, *Klra9*, *Tnfsf10*, *Pik3cg*, *Nras*, *Ptk2b*	*Shc4*, *Shc3*	2.353694
Calcium signaling pathway	<0.01	76	*Prkcb*, *Adora2a*, *Cd38*, *Cysltr2*, *Slc25a5*, *Cacna1b*, *Tacr1*, *Hrh2*, *Plcb2*, *P2rx4*, *Htr7*, *Pde1b*, *Nos2*, *Adcy7*, *P2rx7*, *Sphk1*, *Cysltr1*, *Cacna1f*, *Ptafr*, *Cacna1h*, *Gna15*, *Htr2b*, *Adora2b*, *Bdkrb1*, *Adra1a*, *Plcg2*, *Orai2*, *Ptk2b*, *Cacna1d*	*Ptger1*, *Tacr3*, *Adcy1*, *Grin2c*, *Agtr1b*, *Adra1d*, *Ryr3*, *Pln*, *Adcy9*, *Pde1c*, *Chrm3*, *Grin2a*, *Cacna1s*, *P2rx2*, *Gna14*, *Nos1*, *Adrb3*, *Slc25a4*, *Tnnc1*, *Slc8a3*, *Camk2b*, *Erbb4*, *Adra1b*, *Stim1*, *Ednrb*, *Chrm2*, *Atp2a1*, *Ryr2*, *Plcd3*, *Adcy2*, *Ryr1*, *Phkb*, *Mylk2*, *Plcd4*, *P2rx6*, *Mylk4*, *Vdac1*, *Erbb3*, *Adcy8*, *Atp2b3*, *Slc8a2*, *Tnnc2*, *Cacna1i*, *Camk2a*, *Calml3*, *Phkg1*, *Grpr*	1.971
NF-kappa B signaling pathway	<0.01	48	*Prkcb*, *Vcam1*, *Bcl2a1a*, *Ptgs2*, *Tnfaip3*, *Tnfsf14*, *Zap70*, *Icam1*, *Trim25*, *Malt1*, *Ddx58*, *Nfkbia*, *Il1b*, *Irak4*, *Cd40lg*, *Cflar*, *Plau*, *Gadd45b*, *Tnf*, *Tnfsf13b*, *Tnfrsf11a*, *Ccl4*, *Lck*, *Ly96*, *Myd88*, *Ticam2*, *Lyn*, *Cd40*, *Ripk1*, *Blnk*, *Btk, Bcl2a1d*, *Nfkb2*, *Lat*, *Cd14*, *Syk*, *Tnfsf11*, *Ltb*, *Bcl2a1b*, *Lta*, *Card11*, *Bcl10*, *Plcg2*, *Birc3*, *Traf1*, *Cxcl2*, *Relb*	*Prkcq*	2.240
Chemokine signaling pathway	<0.01	78	*Ccl3*, *Gngt2*, *Ccl20*, *Prkcb*, *Cxcl13*, *Ccr1*, *Vav1*, *Cxcr6*, *Ccr7*, *Cxcr4*, *Pik3r5*, *Cxcl11*, *Gnai2*, *Ccr4*, *Ccr9*, *Ccl22*, *Was*, *Crk*, *Nfkbia*, *Prkcd*, *Ccl6*, *Ccl8*, *Fgr*, *Cxcl10*, *Ccr5*, *Dock2*, *Plcb2*, *Pik3cd*, *Stat1*, *Rac2*, *Adrbk2*, *Ccl2*, *Cxcl1*, *Cxcl16*, *Ccl5*, *Ccl4*, *Arrb2*, *Pik3cb*, *Adcy7*, *Ccl12*, *Stat2*, *Cxcr3*, *Jak3*, *Vav3*, *Lyn*, *Xcl1*, *Cxcr2*, *Cxcr1*, *Ccl9*, *Ccl7*, *Xcr1*, *Src*, *Gng2*, *Ncf1*, *Cxcl3*, *Cxcl9*, *Cxcl5*, *Cxcl2*, *Prex1*, *Gng12*, *Hck*, *Rap1b*, *Ccr8*, *Ccr2*, *Itk*, *Pik3cg*, *Nras*, *Ptk2b*	*Adcy1*, *Ccl24*, *Adcy9*, *Gnai1*, *Adcy2*, *Shc4*, *Cxcr5*, *Adcy8*, *Gnb3*, *Shc3*	1.848
TNF signaling pathway	<0.01	51	*Mapk13*, *Ccl20*, *Junb*, *Vcam1*, *Ptgs2*, *Tnfaip3*, *Pik3r5*, *Icam1*, *Fas*, *Nfkbia*, *Il1b*, *Fos*, *Cflar*, *Cxcl10*, *Sele*, *Pik3cd*, *Ripk3*, *Tnf*, *Ccl2*, *Cxcl1*, *Ccl5*, *Bcl3*, *Cebpb*, *Pik3cb*, *Socs3*, *Map3k8*, *Ccl12*, *Gm5431*, *Ifi47*, *Mmp9*, *Ripk1*, *Casp8, Csf2*, *Atf6b*, *Mmp14*, *Casp3*, *Mmp3*, *Cxcl3*, *Lta*, *Tnfrsf1b*, *Casp7*, *Nod2*, *Mlkl*, *Birc3*, *Traf1*, *Cxcl2*, *Il18r1*, *Il6*, *Pik3cg*, *Il15*	*Mapk12*	2.145

**Table 3:** The degree of hub genes with PPI in the AIA and control groups.

Nodes	Degree	Regulation	Nodes	Degree	Regulation	Nodes	Degree	Regulation
*Cd4*	45	Up	*Fcer1g*	22	Up	*Itgam*	18	Up
*Cd247*	42	Up	*B2m*	22	Up	*Rsad2*	18	Up
*Stat1*	39	Up	*Lcp2*	22	Up	*Fcgr2b*	17	Up
*Syk*	37	Up	*Fcgr3*	21	Up	*Birc3*	17	Up
*Tnf*	34	Up	*Ddx58*	21	Up	*Actr2*	16	Up
*Il1b*	30	Up	*Zap70*	21	Up	*Fcgr4*	16	Up
*Lyn*	29	Up	*Cd44*	21	Up	*Nlrp3*	16	Up
*Ptprc*	28	Up	*H2-Eb1*	21	Up	*Tlr2*	16	Up
*Lck*	27	Up	*Irf7*	21	Up	*Cd74*	16	Up
*Ptpn6*	26	Up	*Ifit3*	21	Up	*Ifih1*	16	Up
*Myd88*	24	Up	*Tyrobp*	20	Up	*Usp18*	16	Up
*Ifng*	24	Up	*Cxcl10*	20	Up	*Fcgr1*	15	Up
*Jak3*	24	Up	*Ifit1*	19	Up	*Nfkbia*	15	Up
*Cd3e*	23	Up	*Vav1*	19	Up	*Ccl4*	15	Up
*Cd3g*	23	Up	*Cxcr4*	19	Up	*Ripk1*	15	Up
*Casp8*	23	Up	*Ccl5*	19	Up			
*Il6*	23	Up	*Casp1*	18	Up			

### Manual acupuncture at ST36 significantly ameliorates inflammatory pain and foot swelling in AIA mice

Our previous work revealed that MA at ST36 had a greater impact on inflammatory pain in AIA rats than electroacupuncture at ST36; hence, in the present investigation, we used MA at ST36 for treatment. First, we evaluated the analgesic and anti-inflammatory effects of acupuncture on the AIA model from the thermal nociceptive threshold, paw swelling, and joint score. Compared with the control group, the PWL of the inflamed paws of the AIA and MA groups decreased significantly after modeling (**Figure 3B**) (*p* < 0.05), and the paw of the afflicted side swelled (**Figures 3C, D**) (*p* < 0.05). The PWL of the MA group was significantly higher than that of the AIA group after the first acupuncture treatment, and this difference was sustained until day 21 (**Figure 3B**) (*p* < 0.05). As illustrated in **Figures 3C, D**, mice in the AIA group developed chronic swelling on the swollen paws and ankle joints on the first day following CFA injection and lasted for 21 days (*p* < 0.05). The paw edema was decreased by day 7 under MA therapy, and the cure persisted through day 21 (*p* < 0.05). The ankle joint structure of the control group was normal, while the ankle joint of the AIA group displayed local inflammatory cell infiltration and granulation tissue hyperplasia (**Figure 3E**). The pathological score of the model group was significantly higher than that of the control group (**Figure 3F**) (*p* < 0.01). Moreover, MA treatment relieved local synovitis of the ankle joint and decreased pathological scores (**Figures 3E, F**) (*p* < 0.05). These findings suggest the anti-inflammatory and analgesic effects of acupuncture in AIA mice by lowering the thermal nociceptive threshold, as well as foot edema and synovial inflammation.

We performed RNA sequencing and DEG bioinformatics analysis of inflammatory joints in the AIA and MA groups to further investigate the molecular mechanism of acupuncture at ST36 alleviating inflammation response.

### Molecular mechanism of manual acupuncture at ST36 in ameliorating inflammation in the AIA model mice based on bioinformatics analysis

**Figure 5:**
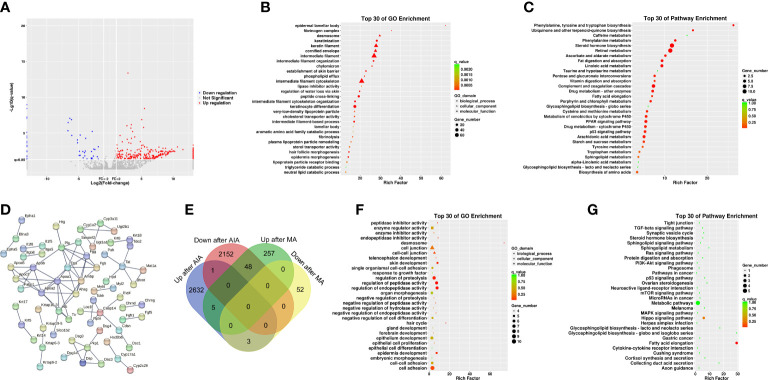
Analyses of the expression and enrichment of genes affected by acupuncture in the joint specimen of mice. **(A)** Scatter plots of differentially expressed genes (DEGs) in the MA and AIA groups. Each probe is represented by a point with red and blue points showing up- and downregulated genes based on log2 FC > 2 as the cutoff. **(B)** GO analyses of DEG results. **(C)** KEGG analyses of DEG results. **(D)** PPI network analyses of DEG results. **(E)** Venn diagram. **(F)** GO analyses of DEGs in the AIA group *vs*. the Con group and the MA group *vs*. the AIA group. **(G)** KEGG analyses of DEGs in the AIA group *vs*. the Con group and the MA group *vs*. the AIA group.

To further investigate the molecular mechanism of acupuncture at ST36 in ameliorating inflammation response, we performed RNA sequencing and DEG bioinformatics analysis of inflammatory joints in the AIA and MA groups. Acupuncture therapy upregulated the expression of 311 genes while downregulating the expression of 55 genes after 21 days (**Figure 5A**), with the majority of the gene alterations being positive. DEGs were mostly enriched in keratinocyte differentiation, peptide cross-linking, keratinization, intermediate filament cytoskeleton organization, epidermis morphogenesis in BP, intermediate filament, intermediate filament cytoskeleton, and keratin filament in CC, cholesterol transporter activity, sterol transporter activity, and lipase inhibitor activity in MF (**Figure 5B**). The KEGG pathway enrichment analysis revealed that DEGs were significantly enriched in pathways involved in steroid hormone metabolism, including steroid hormone biosynthesis, arachidonic acid metabolism, drug metabolism —other enzymes, metabolism of xenobiotics by cytochrome p450 (CYP), drug metabolism —CYP, and peroxisome proliferator-activated receptor (PPAR) signaling pathway. Furthermore, metabolic processes, including retinol metabolism; linoleic acid metabolism; phenylalanine, tyrosine, and tryptophan biosynthesis; phenylalanine, tyrosine, and tryptophan biosynthesis; and fat digestion and absorption were significantly enriched (**Figure 5C** and **Table 4**). The PPI network had 65 nodes and 82 edges (required score > 0.9) (**Figure 5D** and **Table 5**). PPI analyses revealed that acupuncture plays a potential role in lipid metabolism (*Apoa1*, *Apoa2*, *Ambp*, *Alb*, *Apoc3*, *Apoa5*), tissue repair (*Fga*, *Fgg*, *Fgb*), and cell adhesion (*Dsc3*, *Dsg3*). The impact of MA treatment on lipid metabolism was validated by PPI analysis.

**Table 4:** KEGG enrichment of DEGs in the MA and AIA groups.

Pathway	*Q* value	Different genes	Upregulated genes	Downregulated genes	Rich factor
Steroid hormone biosynthesis	< 0.01	11	*Ugt1a1*, *Cyp17a1*, *Cyp2b19*, *Hsd3b6*	*Cyp3a11*, *Cyp2c29*, *Cyp2c44*, *Cyp2d9*, *Ugt2b1*, *Cyp1a2*, *Ugt2b5*	11.714
Drug metabolism —other enzymes	< 0.01	4	*Ugt1a1*	*Ces1c*, *Ugt2b1*, *Ugt2b5*	7.183
PPAR signaling pathway	< 0.01	5	*–*	*Apoa1*, *Apoc3*, *Apoa2*, *Apoa5*, *Fabp1*	5.584
Arachidonic acid metabolism	< 0.05	5	*Pla2g2f*, *Alox12b*, *Cyp2b19*	*Cyp2c29*, *Cyp2c44*	5.145
Metabolism of xenobiotics by cytochrome P450	< 0.05	4	*Ugt1a1*	*Ugt2b1*, *Cyp1a2*, *Ugt2b5*	5.636
Drug metabolism —cytochrome P450	< 0.05	4	*Ugt1a1*	*Ugt2b1*, *Cyp1a2*, *Ugt2b5*	5.468

**Table 5:** The degree of hub genes with PPI in the MA and AIA groups.

Nodes	Degree	Regulation	Nodes	Degree	Regulation
*Apoa1*	9	Down	*Apoa5*	3	Down
*Fga*	8	Down	*Ttr*	3	Down
*Apoa2*	8	Down	*Krtap6-3*	2	Up
*Plg*	7	Down	*Krtap19-5*	2	Up
*Ambp*	7	Down	*Krt5*	2	Up
*Apob*	6	Down	*Efna3*	2	Up
*Alb*	6	Down	*Dsg3*	2	Up
*Fgg*	6	Down	*Dsc3*	2	Up
*Ahsg*	6	Down	*Ugt1a1*	2	Up
*Pkp1*	5	Up	*Ugt2b1*	2	Down
*Casp14*	5	Up	*Cyp17a1*	2	Up
*Tat*	5	Down	*Cdsn*	2	Up
*Apoc3*	4	Down	*Rptn*	2	Up
*Fgb*	4	Down	*Tchh*	2	Up
*Serpinf2*	3	Down	*Mat1a*	2	Down
*DDsp*	3	Up,	*Bhmt*	2	Down
*Cyp3a11*	3	Down	*Fabp1*	2	Down
*Cyp1a2*	3	Down			

In subsequent analyses, we focused on 56 shared DEGs between the AIA and control group, as well as the MA and AIA groups (**Figure 5E** and **Table 6**). Acupuncture potentially exerted an antagonistic effect on RA since it upregulated 48 DEGs that were downregulated after modeling. The DEGs were primarily enriched in peptidase activity regulation, endopeptidase activity regulation, proteolysis regulation, cell adhesion, skin development, and tissue development in BP; desmosome in CC; and endopeptidase inhibitor activity and peptidase inhibitor activity in MF (**Figure 5F**). The KEGG pathway analysis revealed that these integrated DEGs were predominantly enriched in one pathway: fatty acid elongation (**Figure 5G**). The findings suggest that, on the one hand, MA plays a significant role in the regulation of the RA lipid metabolism process, and on the other hand, MA treatment promotes RA tissue repair.

**Table 6:** Co-expressed DEGs among the three groups.

Gene	After AIA	After MA	KEGG pathway	Gene	After AIA	After MA	KEGG pathway
*4732456N10Rik*	Down	Up	–	*Hephl1*	Down	Up	–
*Adgrf4*	Down	Up	–	*Hoxc13*	Down	Up	–
*Aif1l*	Down	Up	–	*Igfn1*	Down	Up	–
*Ankrd55*	Up	Up	–	*Ighv1-54*	Up	Down	–
*Atp6v1c2*	Down	Up	Metabolic pathways	*Klk10*	Down	Up	–
*Cdh19*	Down	Up	–	*Klk11*	Down	Up	–
*Cdsn*	Down	Up	–	*Krt2*	Up	Up	–
*Cgn*	Down	Up	–	*Lrrtm3*	Down	Up	–
*Chrnd*	Down	Up	–	*Luzp2*	Down	Up	–
*Cntn2*	Down	Up	–	*Muc15*	Down	Up	–
*Col19a1*	Down	Up	–	*Peg3*	Down	Up	–
*Col26a1*	Down	Up	–	*Perp*	Down	Up	p53 signaling pathway
*Col7a1*	Down	Up	–	*Pkp1*	Down	Up	–
*Crnn*	Down	Up	–	*Pof1b*	Down	Up	–
*Cst6*	Down	Up	–	*Serpinb11*	Down	Up	–
*Cyp17a1*	Down	Up	Steroid, hormone biosynthesis	*Sln*	Down	Up	–
*Cyp4f39*	Up	Up	–	*Sostdc1*	Down	Up	–
*Elmod1*	Down	Up	–	*Sox2*	Down	Up	–
*Elovl4*	Down	Up	Fatty acid elongation	*Sox2ot*	Down	Up	–
*Epha5*	Down	Up	–	*Spink6*	Up	Up	–
*Fam84a*	Down	Up	–	*Sptlc3*	Down	Up	Metabolic pathways
*Fgf5*	Down	Up	–	*Stbd1*	Down	Up	–
*Fut2*	Down	Up	Metabolic pathways	*Them5*	Down	Up	–
*Gdf5*	Down	Up	–	*Trp63*	Down	Up	–
*Gm26712*	Up	Down	–	*Trpv3*	Down	Up	–
*Gm28653*	Down	Up	–	*Zdbf2*	Down	Up	–
*Gm830*	Down	Up	–	*Zim1*	Down	Up	–
*Gm8797*	Up	Up	–	*RP23-308E4.9*	Up	Down	–

### RNA-seq data validation *via* RT-qPCR

**Figure 6:**
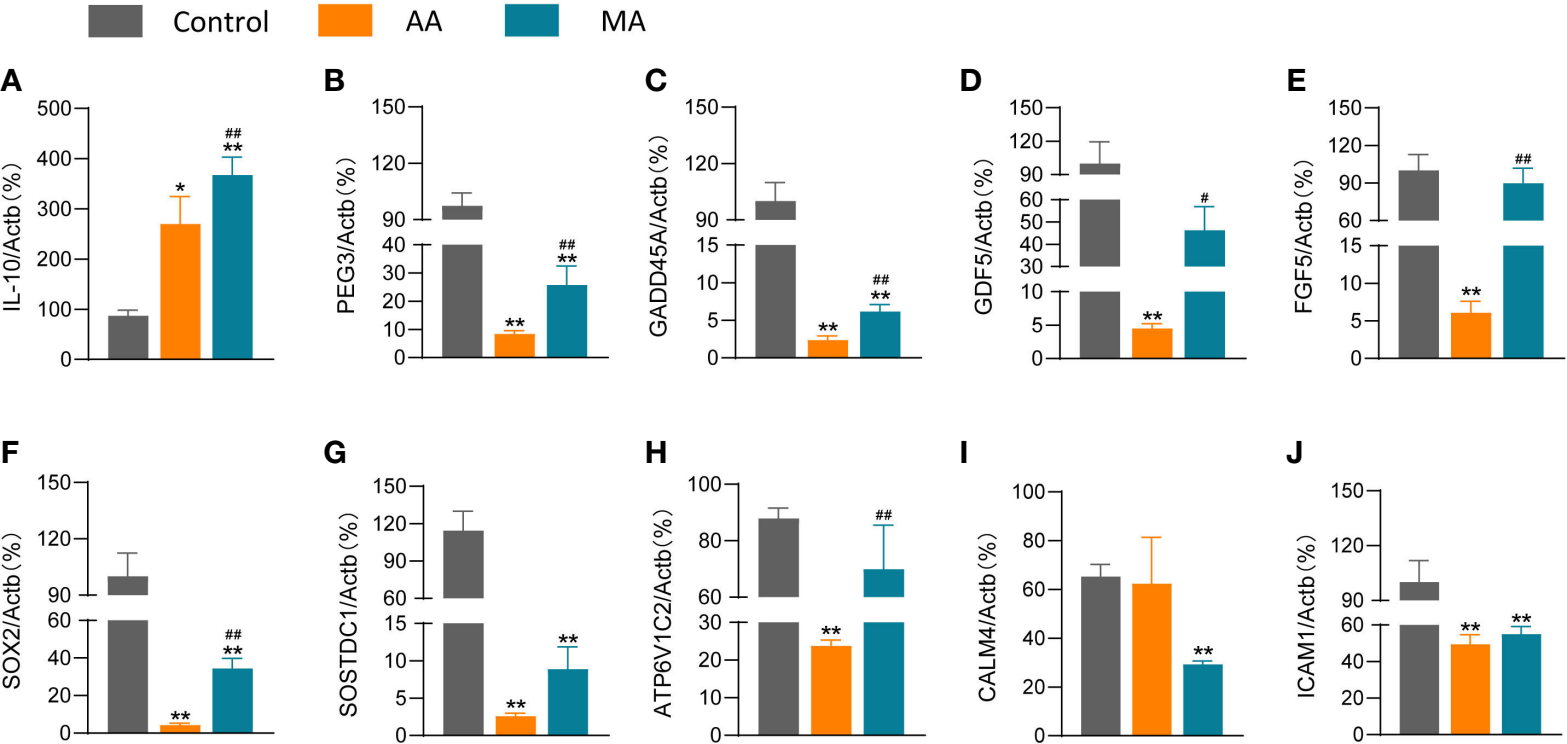
The effects of MA on the expression levels of chemokines and other genes in the joints of AIA mice. The expression levels of *IL-10* **(A)**, *PEG3* **(B)**, *GADD45A* **(C)**, *GDF5* **(D)**, *FGF5* **(E)**, *SOX2* **(F)**, *SOSTDC1* **(G)**, *ATP6V1* **(H)**, *CALM4* **(I)**, and *ICAM1* **(J)** after saline/CFA challenge in mice treated with or without MA (*n* = 6 mice in each group). Data are presented as the mean ± SD. ^*^ *p* < 0.05, ^**^ *p* < 0.01 *vs*. control. ^#^ *p* < 0.05, ^##^ *p* < 0.01 *vs*. AIA.

IL-10 is implicated in the regulation and resolution of inflammation in RA, and inflammation resolution is essential for the repair of cartilage breakdown and bone erosion (17). In this view, we employed RT-qPCR to detect the expression of the anti-inflammatory cytokine *IL*-*10*. The results demonstrated higher *IL*-*10* expression in the AIA group than that in the control group and were further upregulated following manual acupuncture (**Figure 6A**). At the same time, we discovered alterations in *PEG3*, *GADD45a*, and *ICAM1* genes involved in cell cycle regulation, as well as *GDF5*, *FGF5*, *SOX2*, *ATP6V1C2*, *SOSTDC1*, and *CALM4* genes associated with RA bone repair.

A lower expression of *PEG3*, *GADD45A*, *GDF5*, *FGF5*, *SOX2*, *SOSTDC1*, and *ATP6V1C2* was reported in the AIA group than that in the control group (**Figures 6B–H**); however, acupuncture reversed the expression of these genes. *CALM4* and *ICAM1* levels in the MA group did not differ significantly from those in the AIA group (**Figures 6I, J**). However, other genes retained the same trend in the findings of the two tests, and RT-qPCR experiments confirm the reproducibility of the effect of acupuncture on the RA ankle gene expression profile.

## Discussion

RA is characterized by chronic synovial inflammation, imbalance of immune system homeostasis, and dysfunction of a range of immune cell functions and communication networks, which eventually cause irreversible tissue damage. The course of the disease can span decades. The mouse model is extremely significant for investigating the etiology and immunological mechanisms of RA, for validating therapeutic targets, and for developing and implementing new therapeutic strategies. In the present work, we evaluated two experimental models that are routinely used to test prospective treatments for RA, and we hope to uncover targets for acupuncture to improve RA in both animal models.

Intradermal injection of type II bovine collagen caused CIA (18). A secondary immunization is administered 21 days after the primary immunization to ensure significant CIA is induced. The genetic background and immunopathological changes of the CIA model were highly comparable to those of human RA. The key hallmarks are the breach of tolerance and the production of autoantibodies against collagen (19). A few days after the onset of arthritis, the joint is severely devastated by an inflammatory reaction, and the joint remodeling and formation of an erosive pannus tissue exhibit similar histopathology to that found in RA (20). TNF-α, IL-1β, IL-6, IL-10, and transforming growth factor (TGF)-β are among the pro-inflammatory and anti-inflammatory cytokines expressed in the joint (21). DAB/1 mice were used in the present investigation. The joints of CIA mice did not show edema within 21 days of the initial immunization. Some mice in the CIA group began to show slight swelling on day 22 after the second immunization on day 21. The mice began to develop multijoint swelling of both hindlimbs or forelimbs over time, and the swelling location of each mouse varied, and still some mice showed no significant changes. On day 26, the paw swelling significantly differed from that of the control group. The joint swelling and redness of CIA mice peaked on day 32, with the majority of joint swelling in the hindfoot. Despite this, there was a considerable range of paw withdrawal latency among the individual CIA mice, and as a result, no notable variations were observed between the groups. Despite this technical problem, we found that other indicators for evaluating rheumatoid arthritis, such as joint scores, supported the conclusion that the CIA model building was successful. Our findings are consistent with previous research results; significant individual differences in the incidence and distribution of lesions in the CIA model, significant differences in the severity of different foot and paw lesions, and some drawbacks such as low model replication rate limited the effective evaluation of the CIA model. CFA is often injected intradermally into the rat at the tail base or hind paw to initiate arthritis (22). Some characteristics of the AIA model are comparable to those of real RA, such as swelling of the extremities, cartilage degradation, loss of joint function, and lymphocyte infiltration of the joints (23). In the present investigation, BALB/C mice were injected with CFA into the right hind paw to establish the model. The mice developed significant edema and a limp in the ankle joint 24 h after modeling, and their PWL began to deteriorate. This decline lasted until day 21. Furthermore, we observed significant mononuclear cell infiltration and granulomatous inflammatory erosion of the bone, articular cartilage, and ankle interstitial tissue, which is consistent with the histological characteristics of clinical RA. A total of 4,845 DEGs were identified for inflamed ankle joints in the AIA group in comparison with the control group. The majority of DEGs were associated with inflammatory and immune-related pathways such as cytokine–cytokine receptor interaction, CAMs, antigen processing and presentation, and natural killer cell-mediated cytotoxicity. CFA causes a T-cell- dependent autoimmune disease. Bush et al. (24) found that T-cell-induced inflammatory pathways play a significant part in the AIA model. The levels of IL-4, IL-6, MCP-1, and TGF-β successively increase with the progression of joint inflammation. At the same time, we discovered that intracellular signaling pathways, including the calcium signaling pathway, NF-κB signaling pathway, TNF signaling pathway, and Toll-like receptor signaling pathway, are activated in the ankle joint of AIA mice. Inhibition of the aforementioned signaling pathways is thought to have tremendous promise in RA treatment. It is therapeutically utilized for treatment by blocking the pathway linked to the activation of inflammatory gene transcription. Overall, the AIA model met the criteria for autoimmune disease models and was selected as the RA model for subsequent investigations due to its short modeling time, stability, and simplicity in assessing arthritis symptoms such as joint swelling, pain behavior, and joint damage.

The present work revealed that MA treatment suppressed inflammatory symptoms and paw swelling in AIA mice. The PWL in the MA group was significantly higher than in the model group after the first treatment, and afterward, the PWL in the MA group remained relatively stable. The degree of joint swelling on the inflammatory side of the MA group was significantly reduced on day 7. The pathological score demonstrated that acupuncture also decreased inflammatory cell infiltration in the joint. Of note, there was an immediate analgesic effect following acupuncture, which may be connected to the analgesic effect of acupuncture by stimulating local adenosine release (25). In addition, acupuncture-induced pain reduction could be explained by the “pain-gate theory,” which would further activate the multiloop and multitransmitter brain-orchestrated pain modulation and regulate the thalamic and cortical sensory and limbic systems to block pain (26). A recent study discovered that a 21-day MA therapy had potential analgesic and anti-inflammatory effects and decreased the levels of the inflammatory cytokines IL-1 and TNF-α in the damaged paws of AIA rats (27). MA can regulate the balance of macrophage polarization in the affected paw of AIA rats and promote the reduction of the proportion of M1 macrophages and the expression of related cytokines as evidenced by the downregulation of IL-1, IL-18, TNF-α, and IL-6 and the upregulation of M2 macrophages and the expression of cytokines IL-10 and TGF-α (28). Moreover, MA at ST36 was shown to induce the predominance of M2 phenotype macrophages in the inflammatory microenvironment. Due to their great plasticity, macrophages may differentiate into pro- or anti-inflammatory M1 or M2 phenotypes or transdifferentiate into other cell types in response to environmental stimuli and molecular mediators (29). These cells exhibit M2 phenotypes during the tissue repair stage, and the levels of cytokines such as IL-10 and Arg-1 increase, while TNF-α and IL-6 levels decrease (30). We found that *IL*-*10* was increased after MA, as were the other genes that promote tissue repair, including *PEG3*, *GDF5*, *FGF5*, *Atp6v1c2*, *SOX2*, and *Gadd45a*. *PEG3* is an imprinted gene of the TNF/NF-κB signaling pathway and regulates skeletal muscle growth and satellite cell metabolism (31, 32). *Gadd45a* is involved in various processes such as cell cycle arrest, apoptosis, and DNA repair (33). *GDF5* is highly expressed in newly formed cartilage repair tissue after joint injury (34). Intra-articular treatment with recombinant human *GDF5* prevented disease progression and stimulated cartilage repair in a medial meniscal transection model of osteoarthritis in rats (35). These studies provide evidence that *GDF5* plays an important role in the maintenance and repair of articular cartilage. FGF5 is a member of the family of fibroblast growth factors. Clase et al. showed that *FGF5* acts as a mitogen to stimulate the proliferation of mesenchymal fibroblasts and promote the formation of connective tissues such as perichondrium (36). *SOX2* is expressed in immature osteoblasts/osteoprogenitors and is induced by *FGF* signaling, which stimulates osteoblast proliferation and inhibits the differentiation process (37). *ATP6V1C2* is an encoding gene of vacuolar proton pump H^+^-adenosine triphosphatases (V-ATPases). V-ATPases within the resorption lacuna in osteoclasts play a key role in bone resorption by producing an acidic environment (38). GO analysis demonstrated that genes associated with epidermal morphogenesis were significantly enriched after acupuncture. Acupuncture may promote M2 macrophage polarization to achieve anti-inflammatory and tissue repair effects.

Simultaneously, RNA sequencing and bioinformatics analysis were performed on the affected joints of AIA mice treated with acupuncture at ST36. The findings revealed the effects of MA on metabolism, particularly steroid metabolism, including steroid hormone biosynthesis, arachidonic acid metabolism, drug metabolism —other enzymes, metabolism of xenobiotics by CYP drug metabolism—CYP and PPAR signaling pathway, and so on. RNA sequencing results of the MA and AIA groups showed that *Cyp17a1* and *Cyp2b19* gene contents of the cytochrome P450 “Cyp” gene family regulated by acupuncture increased, whereas *Cyp3a11*, *Cyp2c29*, *Cyp2c44*, *Cyp2d9*, and *Cyp1a2* gene contents decreased. CYP regulates inflammation by modulating endogenous substances such as arachidonic acid and epoxyeicosatrienoic acids (EETs) (39). To inhibit inflammation, EETs block TNF-α-induced expression of vascular cell adhesion molecule-1 by inhibiting NF- κB activation (40). Through this similar mechanism, EETs inhibit the activity of different enzymes, including lipoxygenase-5, cyclooxygenase-2, and inducible nitric oxide synthase (41). PPARs potentially regulate gene transcription *via* a ligand-dependent transrepression mechanism by altering other transcription pathways, including NF-κB, signal transducer of activators of transcription, CCAAT/enhancer-binding protein, and activator protein-1. This suppresses the expression of cytokine-responsive genes, limits inflammatory cell recruitment and migration, and lessens vasoconstriction and thrombosis (42). The capacity of CYP to decrease inflammation is boosted by the activation of (PPARgamma) (43). Although corticosteroids are primarily generated by the adrenal gland, they can also be produced in other extra-adrenal tissues such as the immune system, skin, brain, and digestive tract due to the intricate processes involved in steroid synthesis (44). While MA intervention influences genetic modifications linked to steroid metabolism, its precise role and mechanism in joint inflammation in AIA mice warrant further exploration.

## Conclusions

The analgesic and anti-inflammatory effects of acupuncture at ST36 were verified in this investigation, using two types of experimental RA models, AIA and CIA. The AIA model activates the major rheumatoid arthritis pathways as well as antigen immune response pathways. The therapeutic effect of MA on RA is primarily by regulating steroid hormone production, cell metabolism, and tissue repair. Acupuncture may alleviate RA by influencing steroid synthesis and promoting the tissue repair process, which is critical to joint repair. In addition, the hub genes were screened using the PPI network, which verified the results of the GO and KEGG analyses. In addition, the gene expression changes of *PEG3*, *GADD45A*, *GDF5*, *FGF5*, *SOX2*, *ATP6V1C2*, and other genes related to tissue repair and the anti-inflammatory cytokine *IL-10* after MA provide clues for further research on the targets of acupuncture for RA treatment. The main limitation of this study is the lack of experiments to verify the expression level of the putative signal pathway of acupuncture treatment of RA in the afflicted joints. Therefore, more experimental research is warranted to corroborate our findings.

## Data availability statement

The datasets presented in this study can be found in online repositories. The names of the repository/repositories and accession number(s) can be found below: https://www.ncbi.nlm.nih.gov/, 933951.

## Ethics statement

The animal study was reviewed and approved by the Animal Care and Use Committee of Tianjin University of Traditional Chinese Medicine.

## Author contributions

YG and Z-FX conceived the project. HW, Y-QG, and F-MY performed the experiments. YZ and HW analyzed the data. YZ wrote the manuscript draft. Y-QG and FX prepared the figures and the graphical abstract. YG, Z-FX, Y-YL, and S-JW provided administrative, technical, or material support. All authors contributed to the article and approved the submitted version.
